# Esophagojejunal Anastomosis Fistula, Distal Esophageal Stenosis, and Metalic Stent Migration after Total Gastrectomy

**DOI:** 10.1155/2015/839057

**Published:** 2015-04-06

**Authors:** Nadim Al Hajjar, Calin Popa, Tareg Al-Momani, Simona Margarit, Florin Graur, Marcel Tantau

**Affiliations:** ^1^Department of Surgery, Regional Institute of Gastroenterology and Hepatology “Prof. Dr. Octavian Fodor”, Croitorilor Street, No. 19-21, 400162 Cluj-Napoca, Romania; ^2^3rd Surgical Clinic, Iuliu Hatieganu University of Medicine and Pharmacy, Croitorilor Street, No. 19-21, 400162 Cluj-Napoca, Romania; ^3^Training and Research Center “Prof. Dr. Sergiu Duca”, Petre Ispirescu Street, No. 1, 400090 Cluj-Napoca, Romania; ^4^Department of Oncological Surgery, The Oncology Institute “Prof. Dr. Ion Chiricuţă” Republicii Street, No. 34-36, 400015 Cluj-Napoca, Romania; ^5^Department of Intensive Care Unit, Regional Institute of Gastroenterology and Hepatology “Prof. Dr. Octavian Fodor”, Croitorilor Street, No. 19-21, 400162 Cluj-Napoca, Romania; ^6^1st Anesthesiology and Critical Care Clinic, Iuliu Hatieganu University of Medicine and Pharmacy, Croitorilor Street, No. 19-21, 400162 Cluj-Napoca, Romania; ^7^Department of Gastroenterology, Regional Institute of Gastroenterology and Hepatology “Prof. Dr. Octavian Fodor”, Croitorilor Street, No. 19-21, 400162 Cluj-Napoca, Romania; ^8^3rd Medical Clinic, Iuliu Hatieganu University of Medicine and Pharmacy, Croitorilor Street, No. 19-21, 400162 Cluj-Napoca, Romania

## Abstract

Esophagojejunal anastomosis fistula is the main complication after a total gastrectomy. To avoid a complex procedure on friable inflamed perianastomotic tissues, a coated self-expandable stent is mounted at the site of the anastomotic leak. A complication of stenting procedure is that it might lead to distal esophageal stenosis. However, another frequently encountered complication of stenting is stent migration, which is treated nonsurgically. When the migrated stent creates life threatening complications, surgical removal is indicated. We present a case of a 67-year-old male patient who was treated at our facility for a gastric adenocarcinoma which developed, postoperatively, an esophagojejunostomy fistula, a distal esophageal stenosis, and a metallic coated self-expandable stent migration. To our knowledge, this is the first reported case of an esophagojejunostomy fistula combined with a distal esophageal stenosis as well as with a metallic coated self-expandable stent migration.

## 1. Introduction

With every year passing, the incidence of complications following a total gastrectomy is decreasing; literature review shows it occurs in approximately 7–27% of cases [1–4]. Esophagojejunal anastomosis fistula is the main complication of this procedure, with a high mortality rate of around 20%, which represents 30–65% of global postgastrectomy mortality [4–8].

Surgeons tried since the beginning to treat these postoperative leaks and fistulae with drainage and repair, complete parenteral nutrition with no oral intake, and combining these with antibiotics. Nonetheless, this approach had a significant morbidity and mortality rates of up to 60% [9, 10].

Interventional endoscopy comes to the rescue by placing coated self-expandable stents in patients with esophagojejunostomies at the site of the anastomotic leakage. This helps to avoid a complex procedure on friable inflamed perianastomotic tissues [1, 11–13].

The most encountered complication of coated self-expandable stents is stent migration; this according to published literature occurs in up to 28% of cases [14–16]. Management of this condition usually is nonsurgical, being either repositioned endoscopically, waiting for the stent to be eliminated spontaneously through the rectum, or it might even remain in the body if it does not create complications [17, 18]. When the migrated stents create complications, surgical removal is indicated [19].

## 2. Case Report

We present the case of a 67-year-old male patient who was diagnosed with gastric adenocarcinoma intestinal type T2N0M0 (Stage I B), mild microcytic hypochromic anemia, gall bladder lithiasis, essential hypertension grade I, and obesity grade I.

A total gastrectomy with D2 lymphadenectomy with End-to-Side esophagojejunostomy in a transmesocolic Roux-en-Y anastomosis, cholecystectomy, and a feeding jejunostomy were performed.

**Figure 1:**
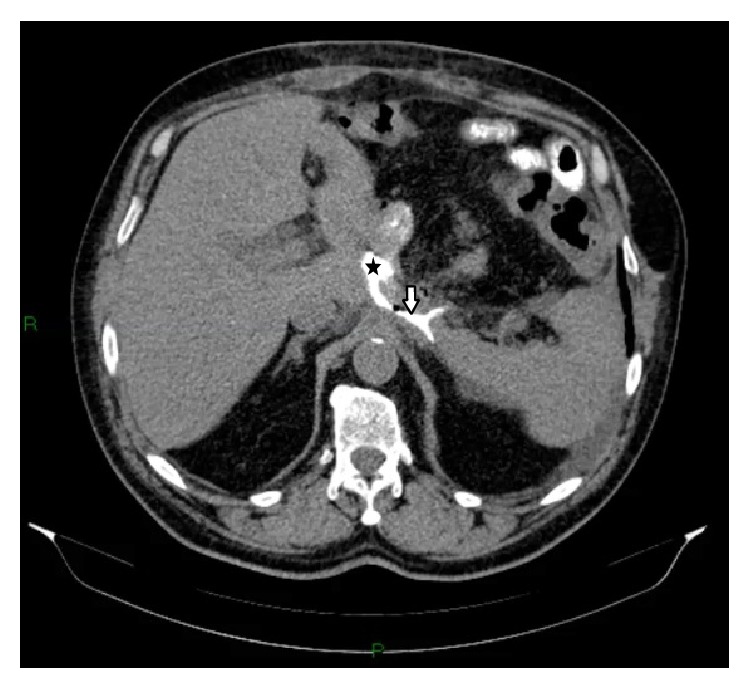
Axial contrast-enhanced abdominal computer tomography (CT): esophageal lumen (star); esophagojejunal anastomotic fistula (arrow).

Postoperative outcome was favorable initially after which his status suddenly worsened, with fever of 38.2°C, loss of appetite, and leukocytosis with neutrophilia. An oral contrast-enhanced CT was performed which showed an esophagojejunal anastomotic fistula (Figure 1).

**Figure 2:**
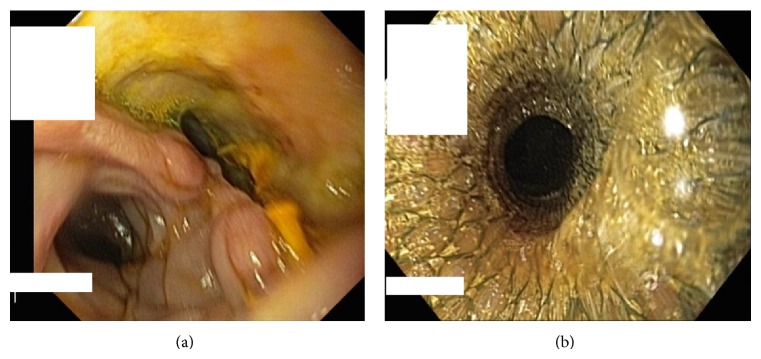
Esophagoscopy: esophagojejunal anastomotic fistula before (a) and after (b) stent placement.

A 28 mm diameter metallic coated self-expandable stent (CSES) was mounted endoscopically on the anastomotic site after which the patient showed a favorable outcome (no reflux complaints) (Figure 2).

**Figure 3:**
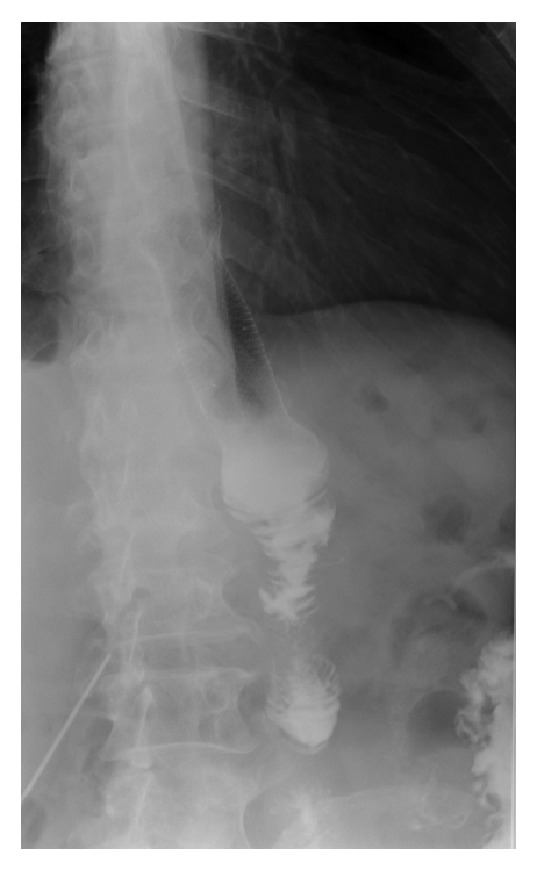
Barium swallow: without extraluminal contrast leakage.

48 hours before discharging the patient and 39 days postoperatively, a barium swallow upper GI radiography was performed which showed good esophageal transit with no contrast material leakage at the anastomotic site (Figure 3).

Histopathological examination of the surgical specimen revealed a gastric adenocarcinoma pT3N0MxL0V0R0 (37 lymph nodes were examined). This puts the patient in the IIA stage of the disease.

The metallic CSES was removed one month after placement, after which the patient started having dysphagia episodes three weeks later. Initially the cause of dysphagia was thought to be a stenosis of the esophagojejunal anastomosis; however, it was further investigated where results showed that the stenosis was situated proximal to the anastomosis, which we interpreted as scar tissue after prolonged local pressure on the esophageal wall due to the presence of the proximal stent tulip.

**Figure 4:**
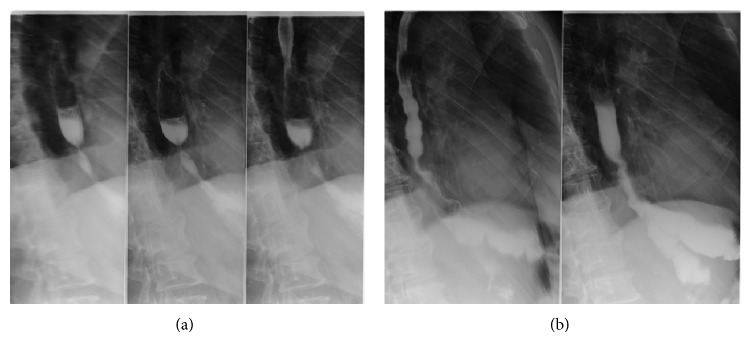
Abdominal X-ray: before (a) and after (b) balloon dilatation.

In the following three-month period he received chemotherapy (FU-FOL protocol) and continued having recurrent dysphagia episodes, for which he was being treated repeatedly by interventional endoscopy with balloon and plug dilation (Figure 4).

**Figure 5:**
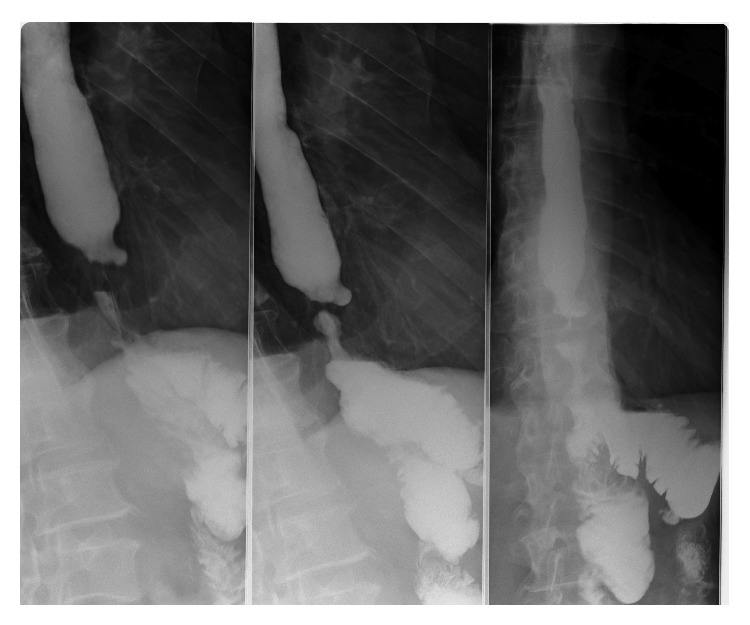
Moderate distal esophageal stenosis before the third stent placement.

To avoid recurrent balloon and plug dilatations, a second (22 mm diameter) CSES was placed two months after the removal of the first one on the distal esophageal stenosis, which was removed one month after placement. A third (22 mm diameter) CSES was placed due to the persisting dysphagia (Figure 5) two months after the removal of the second stent.

One month after the placement of the third stent, the patient presented to the emergency department complaining of diffuse abdominal pain and mixed dysphagia. On physical examination the patient was pale with altered general status and dehydration, and a mass could be palpated in the left paraumbilical region. A fluoroscopy of the abdomen was done, which revealed that the metallic stent has migrated into the jejunum.

**Figure 6:**
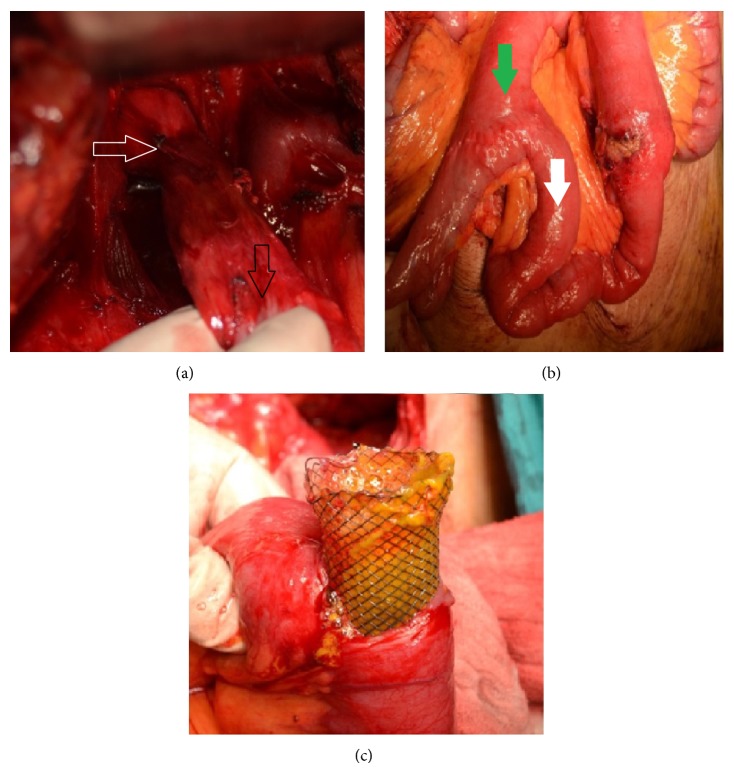
Intraoperative aspects: (a) circumferential distal esophageal stenosis (white arrow), site of esophagojejunal anastomosis (black arrow); (b) migrated stent: direction of migration (white arrow), end-loop proximal small bowel (green arrow); (c) metallic stent into the jejunal lumen.

Correlating the migration of the stent with the increasing intensity of the abdominal pain, a decision of exploratory laparotomy was made, where exploration revealed that the esophageal stenosis was proximal to the esophagojejunal anastomosis, and a migrated metallic stent located just distally to the anastomotic site of the blind loop of the Roux-en-Y procedure performed initially (Figure 6).

**Figure 7:**
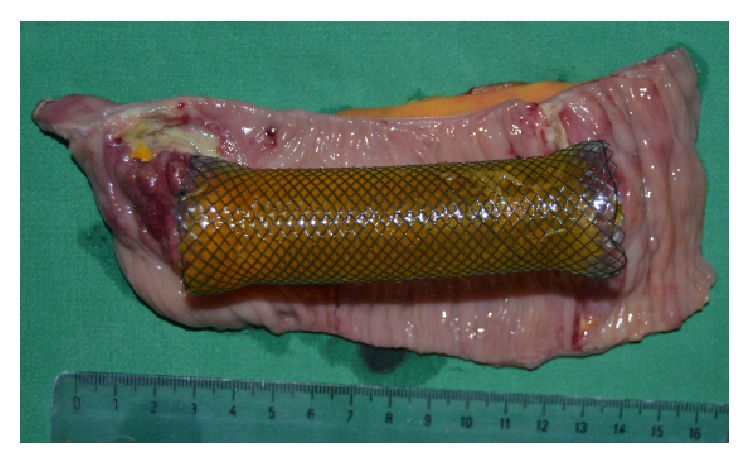
Segmentary enterectomy: postresection specimen and metallic stent.

A distal esophagectomy with a new End-to-Side esophagojejunostomy using a 25 mm circular stapler and an* en bloc* segmentary enterectomy at the site of the stent (Figure 7), with an End-to-End anastomosis, were performed.

Postoperative evolution was favorable, with nutrition per os reestablished after CT evaluation 2 weeks after the last operation, and the patient was discharged a week later.

**Figure 8:**
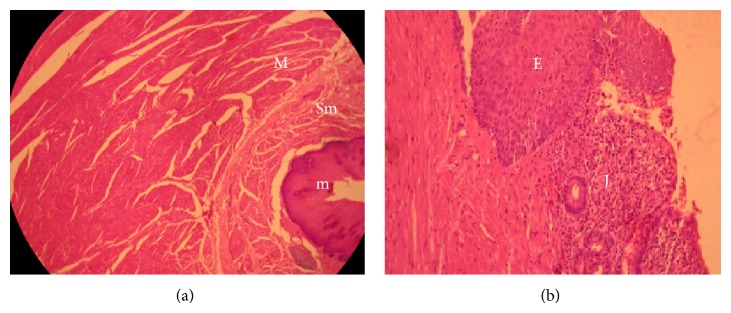
Histopathological findings (H&E ×4) revealing benign distal esophageal stenosis: (a) cross section above anastomotic site (m: mucosa, Sm: submucosa, and M: hypertrophic muscularis propria); (b) esojejunal anastomosis (E: esophageal mucosa, J: jejunal mucosa).

Histopathological examination of the specimens showed that the esophageal stenosis was of benign nature (Figure 8).

The patient was followed up after 6 months after the second intervention, where the esophagojejunal anastomosis was patent with good oral nutrition and gain in weight (6 Kg).

## 3. Discussion

Anastomotic fistula is a complication that is encountered in some patients who underwent gastric surgeries and might lead to poor outcome, poor quality of life, and even life threatening complications. The current management of such cases concentrates on relieving the symptoms, possible complications of the leakage, and providing efficient nutritional support for the patients. This management consists of the drainage of the leaked collections accompanied by long term parenteral nutrition. In more severe cases when the leakage is associated with peritonitis or paralytic ileus, surgical intervention is indicated, despite the possibility of more severe complications due to poor general status of the patient and the high invasiveness of the procedure [20, 21].

In cases of minimal leakage, management consists of discontinuing oral intake and feeding through a jejunal feeding tube or parenterally. Literature shows minimally invasive treatments for esophageal anastomotic leaks like fibrin glue application and endoscopic clipping which showed satisfactory results in some cases, but when the anastomotic leak is large, repeated complex interventions are required to seal these fistulas [22, 23]. Meanwhile endoscopic stenting as a treatment for such fistulas is showing advantages to the patient over immediate surgical reintervention, such as earlier resumption of oral intake, shorter hospitalization, minimal morbidity, better quality of life, and less costs of treatments [21, 24].

As any treatment modality, endoscopic stenting has its complications such as hemorrhage, stent migration, strictures, and perforations. Most migrated stents are removed nonsurgically [19]. However, we believe that in such cases surgical intervention is absolutely necessary if life threatening complications (obstruction, perforation) are imminent.

Benign refractory strictures of the esophagus are the ones where dilation to an appropriate diameter fails and strictures that recur after short periods of time as well as the ones that need continuous dilation [25]. These strictures lower the patient's quality of life due to their possible complications like malnutrition, pain, or perforation [26]. Many techniques are available for treating such strictures, out of which is temporary stenting. Stenting is an appealing approach as the placed stent is performing the dilation function for the complete period of placement, and it can be removed when needed. Nonetheless, temporary stenting must be used in carefully chosen patients as it has a high rate of complications. These temporary stents are useful tools in restoring the lumen of the esophagus until the patient's status or treatment plan allows surgical intervention [27].

Reintervention in our patient had a particularity that it had two purposes: to resolve the imminent complication of stent migration and the distal esophageal stenosis.

The multidisciplinary treatment, interventional endoscopy, and surgery were complementary approaches as interventional endoscopy managed the surgical complication being an esophagojejunal fistula, and later surgery for managing the endoscopic stenting complication being the distal esophageal stenosis and stent migration.

For this case, endoscopic stenting seemed to be the most appropriate immediate management for the esophagojejunostomy fistula. Operative management for the distal esophageal stenosis would have delayed chemotherapy, whereas stenting enabled chemotherapy administration as early as possible and offered the patient a better quality of life during this phase of treatment as well as avoiding repeated balloon and plug dilatation sessions. Taking into account that the stenosis before the third stent mounting was moderate, it might have contributed to the migration of the stent. When the patient's symptoms indicate an upcoming complication of a migrated stent, we consider the operative removal an essential step for a better prognosis.
